# Embedding health literacy research and best practice within a socioeconomically and culturally diverse health service: A narrative case study and revised model of co‐creation

**DOI:** 10.1111/hex.13678

**Published:** 2022-11-29

**Authors:** Danielle M. Muscat, Dana Mouwad, Kirsten McCaffery, Dipti Zachariah, Lyn Tunchon, Julie Ayre, Don Nutbeam

**Affiliations:** ^1^ Sydney Health Literacy Lab, School of Public Health, Faculty of Medicine and Health The University of Sydney Sydney New South Wales Australia; ^2^ Western Sydney Local Health District, Integrated and Community Health Health Literacy Hub Sydney New South Wales Australia; ^3^ Western Sydney Local Health District, Integrated and Community Health Multicultural Health Sydney New South Wales Australia; ^4^ Western Sydney Local Health District, Integrated and Community Health Child and Family Health Sydney New South Wales Australia; ^5^ School of Public Health, Faculty of Medicine and Health The University of Sydney Sydney New South Wales Australia

**Keywords:** co‐creation, health literacy, health research systems, knowledge production, research translation

## Abstract

**Background:**

Health literacy interventions and research outcomes are not routinely or systematically implemented within healthcare systems. Co‐creation with stakeholders is a potential vehicle through which to accelerate and scale up the implementation of innovation from research.

**Methods:**

This narrative case study describes an example of the application of a co‐creation approach to improve health literacy in an Australian public health system that provides hospital and community health services to one million people from socioeconomically and culturally diverse backgrounds. We provide a detailed overview of the value co‐creation stages and strategies used to build a practical and sustainable working relationship between a University‐based academic research group and the local health district focussed on improving health literacy.

**Results:**

Insights from our experience over a 5‐year period informed the development of a revised model of co‐creation. The model incorporates a practical focus on the structural enablers of co‐creation, including the development of a Community of Practice, co‐created strategic direction and shared management systems. The model also includes a spectrum of partnership modalities (spanning relationship‐building, partnering and co‐creating), acknowledging the evolving nature of research partnerships and reinforcing the flexibility and commitment required to achieve meaningful co‐creation in research. Four key facilitators of health literacy co‐creation are identified: (i) local champions, (ii) co‐generated resources, (iii) evolving capability and understanding and (iv) increasing trust and partnership synergy.

**Conclusion:**

Our case study and co‐creation model provide insights into mechanisms to create effective and collaborative ways of working in health literacy which may be transferable to other health fields in Australia and beyond.

**Patient and Public Contribution:**

Our co‐creation approach brought together a community of practice of consumers, healthcare professionals and researchers as equal partners.

## 1 INTRODUCTION

Improving health literacy is an international policy priority, grounded in evidence that links lower health literacy with poorer health outcomes.^1^ Most health literacy policies recognize that the responsiveness of the health system needs to be improved^2^ and many identify the need for greater implementation of evidence‐based practices and research.^3^ However, research outcomes are not routinely or systematically implemented within healthcare systems.^4^, ^5^, ^6^, ^7^ This is true across all health research domains, including for research on health literacy.^8^, ^9^ This failure to systematically translate, implement and deliver evidence‐based improvements in outcomes for patients and the community has attracted attention to the way we conduct health services research, its perceived relevance and the potential for practical implementation by health professionals and health organizations. This, in turn, has led to advocacy for more effective and consultative partnerships at every stage of the research process, from initial idea generation to implementation.^10^, ^11^

This approach to the collaborative generation of knowledge by academics working alongside stakeholders from other sectors is often referred to as co‐creation.^12^ The concept of co‐creation is grounded in the belief that proactive linkage and exchange builds bridges between researchers and the intended users of research (health professionals, patient and community members), and develops the mutual trust on which successful collaboration depends.^12^ Answering research questions that have been generated through partnership can lead to interventions that are closer to consumer needs and preferences, are ‘owned’ by health professionals and more likely to be sustained.^12^ In this way, co‐creation is a potential vehicle through which to accelerate and scale‐up the implementation of innovative research and support longer‐term sustainability of a change in practice.

Although there is a growing literature about co‐creation and its contribution to research translation, there remain relatively few working examples. In the domain of health literacy, a small number of studies report on the ‘co‐creation’ of solutions to improve the design and navigation of health services and written materials with patients and consumers.^13^, ^14^, ^15^ However, these examples are narrowly focused on one aspect of health service delivery, and often fail to engage the full spectrum of end users of the research including clinicians, health service managers and other key stakeholders within healthcare systems. These existing studies also appear to be researcher‐driven with consumer involvement often limited to market research and testing. Few exhibit the key features of co‐creation including involvement and input from the strategic partners throughout the entire research journey (i.e., from the development of the research questions to the implementation and evaluation phases).^16^

This paper presents a model developed over a 5‐year period to build a practical and sustainable working relationship between a University research group and a local health district working across clinical and community services and focussed on improving health literacy (i.e., the ability of individuals to gain access to, understand, appraise and use information in ways which promote and maintain good health^17^). Previous research has consistently shown that low health literacy has a negative impact on healthcare access,^18^ physician–patient communication,^19^ medication adherence^20^ and effective healthcare use^18^; and that organizational factors including clinical communication have a major role in easing or complicating health for people with limited health literacy. Communication between clinicians, patients and carers is a core business in healthcare systems but is often done poorly.^21^, ^22^ To address this, we have sought to develop a working relationship to support enhanced clinician communication, organizational health literacy responsiveness and improved consumer health literacy that is led by health system priorities, engages patients, consumers and health staff, and is based on high‐quality research.

## 2 METHODS

### 2.1 Setting and context

Here we describe an example of the application of a co‐creation approach to improve health literacy in a local health district that provides hospital and community health services to one million people in a culturally and economically diverse community in Sydney, Australia. Following the case study approach of Greenhalgh et al.,^12^ we present this case in narrative form. We provide a detailed overview of the value co‐creation stages and strategies to allow others to consider and apply our learnings in different health system contexts. This narrative case study also provides a basis from which we can compare existing conceptual models with practical experience in a real‐world context.^23^

#### 2.1.1 Policy context

Over the past 5 years in Australia, there have been consistent policy statements advocating a more systematic approach to embedding interventions to improve health literacy within the healthcare system. This has been justified as a priority for health, social and economic reasons.^24^, ^25^ The Australian Commission on Safety and Quality in Health Care mandates improvements in health literacy for clinical safety and social justice reasons.^26^ To this end, the Australian government has implemented new regulatory requirements for health literacy through prescribed criteria and actions for health literacy in the revised National Standards (2019) for health organizational accreditation. All public and private hospitals are required to be accredited to the Standards, with a 3–4 year accreditation cycle.^27^ The 2020–2025 National Health Reform Agreement similarly focuses on ‘empowering people through health literacy’ with an emphasis on person‐centred health information and support to enable consumers to manage their own health and engage effectively with health services.^28^ Given this context, strategic priorities for local health districts often reflect the need to improve communication and help people to better understand their health and manage their care.^29^

#### 2.1.2 Geographical and social context

Western Sydney Local Health District (WSLHD) is 1 of 15 local health districts in the New South Wales (NSW) health system. WSLHD has approximately 12,000 staff members and delivers services to almost one million residents in Sydney's west. It has the highest urban indigenous population in Australia, 47% of residents were overseas‐born, and one in two speak a language other than English at home.^30^ WSLHD is one of the state's fastest growing areas with more than 1.3 million residents estimated by 2031, with a disproportionate increase in people aged 70 years or over.^30^

### 2.2 Co‐creation steps

Given the increasing policy emphasis and growing national interest in health literacy and the diverse needs of western Sydney, the idea of developing a ‘Health Literacy Hub’ to provide a consolidated support service for staff emerged in mid‐2017. A senior health manager identified the need for a more systematic approach to addressing health literacy in the health district and reached out to established contacts at a local university. The initial team was attracted to a co‐creation approach which brought together a community of practice of healthcare professionals and consumers with an interest in improving health literacy in western Sydney to form the Health Literacy Hub alongside an established academic health literacy team (the Sydney Health Literacy Lab; https://sydneyhealthliteracylab.org.au/). This was seen as a mechanism by which many of the local priorities and national strategic imperatives could be met. Although it was recognized that health literacy cannot compensate for health inequities created by the unequal distribution of opportunity and resources in societies, we were motivated by the belief that it is possible to optimize the contribution health literacy makes in mediating the causes and effects of established social determinants of health.^31^

The partnership also provided a focus and dedicated resource for testing, adaptation and implementation of health literacy interventions. By bringing people with a common interest in health literacy together, we hoped to move from a previously siloed approach in addressing the issue, to a multidisciplinary, collaborative model of working. This approach could control variability and improve service delivery effectiveness and research output by leveraging the combined resources of a university and a local health district. Co‐creation was seen as a mechanism to create a ‘win more–win more’ environment for health literacy research and practice.^16^

#### 2.2.1 Establishing the strategic direction for the Hub

We invited internal and external stakeholders (including service users, primary and secondary care providers, health services managers and health department policy‐makers) to determine the strategy and priorities for the Health Literacy Hub. Meetings involved a structured workshop format, drawing on elements of the Nominal Group Technique.^32^ Each stakeholder was invited to state their priorities for the Hub, which were each recorded and then discussed as a group. The purpose of this discussion was to allow stakeholders to clarify, elaborate, defend or dispute the items and to add any new priorities that emerged from the discussion. Priorities were grouped into three broad themes presented in Box 1. It was agreed that all Hub activities would be anchored in these priorities and aligned to the local health district's priorities, as determined jointly with our stakeholders.

Box 1 **Health Literacy Hub priorities**

1

(1) *Build staff capacity*: Provide practical assistance to clinicians to better understand the communication needs of their patients; and equip them with evidence‐based methods and tools to optimize the impact and effectiveness of communications with patients and consumers.
(2) *Create a health literate organization*: Establishing systems and organizational structures that enable and reinforce effective patient and public communication, including health services navigation and physical wayfinding.
(3) *Provide public resources, tools, support and advice* to assist patients, their carers and families to communicate and connect in a meaningful way to the broader health system; specifically supporting them to understand and utilize the information provided and make informed decisions.

#### 2.2.2 Developing shared management systems: Governance, leadership, resourcing

Successful and sustainable partnerships require resources.^33^ Having identified priorities for the Hub, we were better placed to secure funding to support our partnership. We were able to successfully position the Hub as an important resource supporting core Local Health District objectives in improving clinical quality and safety, and enabling it to meet current and future requirements for institutional accreditation. This alignment with the core purpose was important in securing executive support and subsequent resource allocation.

Initial funding was provided for 4 years to support a ‘Director of Strategy and Operations’ position for the Hub and a Senior Academic Advisor. The Academic Advisor was a senior University academic embedded in the local health system, with previous experience working in both health and academic sectors. We recognized that partnerships need boundary‐spanning leaders who understand and appreciate partners' different perspectives, can bridge their diverse cultures and are comfortable sharing ideas, resources and power.^33^ Initially, the Academic Advisor prioritized building good working relationships, trust and openness among partners; ensuring that our health services partners had access to the best available evidence to support them in thinking and working differently and mobilizing the resources needed to support the development of the partnership of the university, Local Health District and external sources. These early actions provided a shared sense of purpose on what the Health Literacy Hub partners could accomplish together, and how their joint work would benefit not only the community but also each of them individually.

The Director of Strategy and Operations' role was to work on transformational change and to support awareness, engagement and increased capacity of healthcare staff to improve health literacy. They too acted as a boundary‐spanner working with university colleagues in the development of a supporting programme of health literacy research. The Director had connections to people, organizations and groups—including target populations, political decision‐makers, government agencies, private sector funders and other partnerships in the community—as well as ‘convening power’ to bring people together for meetings and other activities.^33^ Unlike more bureaucratic forms of management, which are often rigid and structured to control what people do, we endeavoured to have a management approach that was more flexible and supportive particularly given that we were engaging with health staff in established roles who were employed centrally rather than through Hub funding.^33^

These two positions were bolstered by early success in attracting funding for a health literacy Postdoctoral Research Fellow who would act as an important day‐to‐day point of connection between the health services Hub and the university Lab. The Research Fellow played a strategic role in brokering academic evidence and knowledge related to health literacy and bringing it into the Hub and working directly with health district staff and consumers to enable health literacy research.

#### 2.2.3 Building a community of practice through a network of engagement platforms

To bring health staff, consumers and researchers together, we sought to develop a Community of Practice. Wenger^34^ described Communities of Practice as building blocks of a collective learning system. They are dynamic social groups bound by a common concern or passion and a desire to learn how to improve their practice. Communities of practice differ from other forms of organization in several ways. They are not designed to deliver a specific product or service or to complete specific projects or tasks in the same way that a formal work group or department would be.^35^ Communities of practice also differ in that membership is self‐selected, and that passion, commitment and identification with the group's expertise holds the group together rather than specific project milestones.^35^ In this way, the Health Literacy Hub was developed to be a point of connection for researchers, health staff and consumers to share information, solve problems and drive innovation in health literacy.^34^ It was a way of aligning people with shared values and commitment to health literacy.

To build the Community of Practice, we strategically designed a network of engagement platforms. See Table 1. These included the development of a Health Literacy Hub website, seminar series and Community of Practice mailing list. Hub staff and university academics were also involved in a number of one‐on‐one consultations and targeted health literacy training initiatives with Local Health District staff. The goal of this broad engagement strategy was to build interest in health literacy, support continuing professional development for health staff and iteratively develop and expand the circle of stakeholders engaged with the Health Literacy Hub.

**Table 1:** Engagement platforms used to build and engage a community of practice

Initiative	Description
Health Literacy Hub website	The Health Literacy Hub website built during 2018 is designed to largely support staff development, exchange of ideas and information, and to facilitate access to useful, best practice health literacy tools and resources. To this end, the website is organized on three levels:
	Level 1—Publicly accessible, including information about health literacy and the Health Literacy Hub, and external links to support consumers to find health information, access health services etc.
	Level 2—Accessible through registration to health professionals and the academic community, providing access a wide range of educational materials, practical tools and advice on health literacy.
	Level 3—Accessible to Western Sydney Local Health District staff only, including access to an online system to support staff in developing health literate consumer information.
Health literacy seminar series	The bi‐monthly seminar series introduces health literacy concepts (e.g., e‐health literacy), evidence‐based health literacy interventions and practices (e.g., teachback), and relevant policy (e.g., the Australian National Standards) in an accessible manner. The annual programme is formulated collaboratively by researchers, health staff and consumers, and seminars are generally co‐presented by an academic and healthcare professional.
Community of practice mailing list	An electronic mailing list was developed to facilitate the distribution of information to Community of Practice members, such as information about upcoming seminars and available health literacy resources. The mailing list is also intended to foster interactivity between members, such as through moderated problem solving.
Meetings and consultations	The Hub has strategically engaged with people embarking or already undertaking health literacy initiatives for consultation, advice, the shaping of ideas or proposals and determining ways of engaging more directly with researchers. This has been facilitated through co‐location of university and health staff within the Local Health District.
Targeted training	The Hub has also led formal, targeted training in health literacy with over 190 clinical (Allied Health; Child and Family Health Nursing) and preclinical (Pharmacy; General Practice) staff and students to date. Building ‘capability ecosystems’^12^ in this way is intended to improve access to research and evidence‐based health literacy practices, as well as expand the circle of stakeholders engaged with research.

### 2.3 Co‐creation in research practice

Having in place jointly determined priorities, continuous learning opportunities and an active Community of Practice provided a platform to facilitate the implementation of evidence‐based health literacy practices in the local health district as well as to bring together a range of health staff, consumers and researchers to co‐create research projects together from the outset. Early research partnerships were often consultative, with healthcare staff seeking feedback and advice on decisions or analyses related to health literacy research which had already been conducted (see Box 2 Case Study 1, e.g.). While these early partnerships were important for relationship building, they were ultimately missing core elements of co‐creation—namely, collaboration from the outset to develop research questions, co‐design research activities and plan and implement evaluation frameworks.

Box 2. **Case studies**

1

**Table:** 

Case study 1—‘Relationship building’ Integrated and Community Health	Case study 2—‘Partnering’ Allied Health
Over a 4‐year period, Integrated and Community Health in Western Sydney Local Health District delivered the Stanford Chronic Disease Self‐Management Program (CDSMP^36^) to 486 people living with one or more chronic disease, and assessed health literacy pre‐ and postintervention. The Integrated Chronic Care Program Manager partnered with the Health Literacy Hub to analyse the data from this project. Outcomes of value were achieved through partnership; our analysis identified statistically significant improvements across all domains of health literacy,^37^ and provided evidence of programme effectiveness to support continuation of its application for patients with chronic conditions across the Local Health District. Healthcare staff, students and researchers also co‐authored a research publication in a journal special issue.	Before the development of the Health Literacy Hub, the WSLHD Allied Health Research Group conducted a cross‐sectional survey of health literacy in outpatient allied health clinics. Employing a strategic approach, the Hub was able to partner with the Allied Health Research Group in the analysis of the data from their survey.^38^ Building on this initial collaboration, allied health staff became integral members of the Hub, and continued to work with researchers to develop, implement and evaluate a targeted health literacy training programme for allied health professionals in western Sydney.^39^ Allied health staff in this partnership co‐presented a Hub seminar on health literacy measurement in 2018.

Over time, research collaborations have moved away from consultation towards models of partnering and co‐creation where healthcare staff and researchers have worked together from the outset to frame locally relevant research questions, create research designs that reflect ‘real‐world’ environments and commit to both implementing research as well as utilizing and embedding findings in the broader health service delivery community. For example, Case Study 2 (Box 2) reflects an evolving research partnership in which early consultation with members of the Community of Practice built interest, awareness and knowledge of health literacy and opened up future possibilities for more integrated research partnerships with this group.

### 2.4 Facilitators of co‐creation

As research partnerships have evolved over time, we have identified key facilitating factors for co‐creation including identification of local champions, co‐generated resources, increasing trust and partnership synergy, and evolving capability and understanding. These are discussed in turn below and supplemented by Table 2 through the example of the Parenting Plus project that has brought together researchers, health staff and consumers to embed health literacy training into child and family health services in western Sydney.

(1) Local champions—The activities of the Health Literacy Hub have been strengthened by the identification of local champions who have been proactive in advocating for cultural change and facilitating partnership projects across the District—both directly as partners themselves and indirectly through outreach activities which they have mediated.^33^ In the early stages of developing the Health Literacy Hub, the Director of Strategy and Operations identified staff with natural leadership characteristics and prior commitment to improving health literacy, and facilitated meetings and engagement.
(2) Co‐generated resources—Financial and in‐kind resources are the basic building blocks of co‐creative interaction and research. Ongoing research collaborations have been facilitated by co‐generated research funding—allowing dedicated human and material resources to drive specific research projects. Funding applications have necessarily involved both health staff and academics (with alternating leads based on the funding scheme), and have strategically included both direct research costs and budget to build capacity for research within the district through participatory approaches (e.g., clinical staff secondments).^41^
(3) Evolving capability and understanding—As researchers have become increasingly engaged with the Local Health District, we have seen bidirectional learning and knowledge gain for different stakeholders, including an evolving understanding of different values, needs, and ways of working in research and clinical practice. This has been facilitated through deliberate actions such as the co‐location of research and clinical staff and the prioritization of clinical staff secondments to the Hub.
(4) Increasing trust and partnership synergy—There has been a sustained commitment to building relationships of trust between researchers and communities engaged with the Health Literacy Hub. Both within and across projects, we have seen increasing partnership synergy (i.e., synergy that arises from collaboration among members of diverse knowledge, perspectives and cultures) as researchers and health professionals have worked together over time. The synergy of collaboration is manifested in the increasing number of co‐created research projects across the District, as well as a shift in the point of engagement; rather than working with university academics at the analysis stage, health staff and researchers have increasingly come together at the earliest stages to identify problems, generate solutions and consider practical, culturally appropriate methodological approaches.

**Table 2:** Four key facilitating factors of co‐created research in the Parenting Plus project

Key facilitating factor	Demonstration in the Parenting Plus project
Local champions	Researchers and Western Sydney Local Health District Child and Family Health staff first came together in 2018 in an initial meeting facilitated by the Hub Director. The Program Lead of Child and Family Health had previous experience working on health literacy projects, advocated for health literacy and agreed to partner in pilot testing the programme across six sites. Initial stages of the project were also enabled through strategic engagement with the Program Lead of Multicultural Health who championed health literacy, the Parenting Plus programme and the co‐creation approach across the District. Our local champions connected us with consumers (new parents) who also became partners in the development and adaptation of the Parenting + materials, ensuring that the programme was developed in consultation with multicultural communities from the health district.
Co‐generated resources	Initial funding for the piloting of the Parenting Plus project in western Sydney was awarded—on a competitive basis—from the Local Health District's Research and Education Network and the Primary Health Network. This funding was strategically allocated to direct research costs related to roll‐out of the pilot programme and the secondment of a Child and Family Health Nurse to work directly in the Health Literacy Hub for the duration of the pilot. Successful piloting informed a larger funding application for a randomized trial of Parenting Plus, awarded in 2021.
Evolving capability and understanding	Evolving capability of researchers and health staff was achieved through the secondment process which enabled health staff and researchers to work directly together on the Parenting Plus project over a 10‐month period.^40^ Formal and informal interactions between team members during this time helped us to appreciate one another's worldviews, priorities and ways of working. Health staff were also involved in research capacity building and transferable skills training in data collection and analysis.
Increasing trust and partnership synergy	Increasing partnership synergy was evidenced by modifications to the Parenting Plus programme made postpilot which better reflected the perspectives and priorities of community stakeholders, including the target population (new parents) and health staff. Researchers (*n* = 2) and health staff (*n* = 2) worked together to analyse and interpret feasibility study data and identify necessary modifications to programme content which was iteratively reviewed by managerial health staff (*n* = 3) and patient partners (new parents; *n* = 3) in a series of workshops and follow‐up correspondence.

Increasing trust and partnership synergy between researchers and health district staff has also served to strengthen collaborations with consumers and the broader community. The District's—and, in particular, our local champions'—strong ties to the community have strengthened the capacity of the Hub to access and involve community members in the co‐creation of health literacy research. In addition to Parenting Plus, another recent example of this was the rapid mobilization of staff and consumers in Western Sydney and two adjoining health districts to co‐design and conduct the largest Australian COVID‐19 survey of people who speak a language other than English at home.^42^, ^43^

## 3 RESULTS: A (REVISED) MODEL OF CO‐CREATION

**Figure 1:**
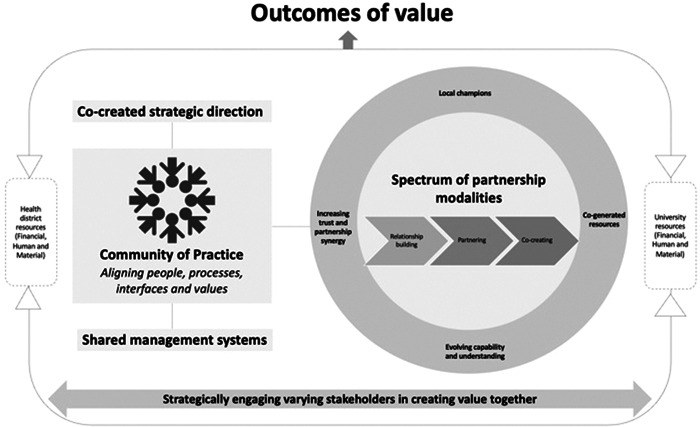
Revised model of co‐creation

Insights from our experience over a 5‐year period have led to a refined understanding of how co‐creation is facilitated in practice, as summarized in Figure 1. At the core of the partnership has been a common vision oriented to improving health literacy and delivering outcomes of value for academics, health staff and consumers combined with the engagement of each stakeholder group at every stage.

Our revised model for research co‐creation builds on previous work, but advances from this foundation by focussing on practical stages, strategies and structures to build research partnerships and work towards co‐creation in real‐world health services and health systems. Three key elements of differentiation from existing conceptual models of co‐creation^12^, ^16^ are outlined below.

(1) Our revised model incorporates a practical focus on the structural enablers of co‐creation. The centrality of the Community of Practice, for example, reflects the key role that the iterative expansion of the Community of Practice played in enabling us to reach a broad range of consumers and health providers and to grow the Hub's presence within the district. This was key to facilitating co‐creation across multiple settings and projects.
(2) By including a spectrum of partnership modalities (spanning relationship‐building, partnering and co‐creating), our model acknowledges the evolving nature of research partnerships and seeks to realistically reinforce the flexibility and commitment required to achieve meaningful co‐creation in research. We employed a range of connected strategies and invested the necessary time and resources to develop capability, trust and understanding between researchers, frontline health workers and consumers. Importantly, this foundational work enabled research partnerships to evolve over time.
(3) Finally, we have brought together literature related to co‐creation^12^, ^16^ and partnership synergy^33^, ^41^, ^44^ to depict key facilitators of research and collaboration including (i) local champions who advocated for health literacy and institutional collaborations and had the ability to influence change regardless of organizational position, (ii) co‐generated resources, particularly from external sources, (iii) evolving capability ecosystems and understanding and (iv) increasing trust and partnership synergy.

### 3.1 Barriers and challenges

While the process of developing the Health Literacy Hub has informed the above model of co‐creation, we have also faced challenges and barriers in the establishment and maintenance of this collaborative partnership which warrant attention. Foremost, the experience of bringing together the Hub and the Lab has highlighted the flexibility and commitment required to achieve meaningful co‐creation in research. Processes for partnering in the Health Literacy Hub have necessarily been dynamic and evolving, and this has required significant investments of time over and above other models for conducting health research. Given the inherent time commitments, one of the greatest threats to the maintenance of Hub relationships has been the turnover in staff, senior managers and executives in the health district. For example, the Lead of Child and Family Health who co‐led the development and feasibility testing of the Parenting Plus programme retired, as has the Child and Family Health nurse seconded to co‐design programme content. There has also been a turnover of Chief Executives and several Executive Directors since the establishment of the Hub. To maintain the momentum and stability of the Health Literacy Hub despite such turnover, we continue to engage broadly with staff at all levels of the organization and externally to sustain relationships and continually build new ones.^45^ To date, we have had over 70 consultations with different clinical services in the Local Health District, members of senior management and district executives and external stakeholders including state health services, other local health districts, councils and consumer organizations. An additional challenge has related to ongoing funding and capacity. While we have been successful in obtaining project‐specific funding through grant applications, there is an ongoing need for designated administrative and support staff to maintain engagement platforms (e.g., the Hub website) and capacity‐building initiatives within the District. Funding for such roles has been harder to secure on a sustainable basis.

### 3.2 Outcomes and future directions

To date, key outcomes and achievements relate to reach and the scope of collaborative activities. There are currently over 1300 members of the Community of Practice and 11 completed or ongoing research projects which have quite literally ranged across the lifespan from early childhood/parenting education, through chronic disease management, to end‐of‐life decision‐making. The Lab/Hub collaboration has generated $1.9 million in research project funding, >15 jointly authored research outputs and has been linked to organizational‐level improvements in health communication.^46^ Moving forward, the monitoring and evaluation of Hub research outcomes is an intentional focus, to ensure that efforts are recorded and recognized for their value to both the academic, health and broader communities and consumers. We are also seeking to develop more comprehensive and systematic models for engaging with consumers, patients and carers across all collaborative projects.

## 4 DISCUSSION

The Health Literacy Hub represents a rare form of collaboration between hospitals, healthcare services, communities and health literacy researchers, which has evolved to develop innovative, practical and scalable health literacy interventions. This research ‘laboratory’ has enabled us to develop relevant, contextualized research questions and undertake applied research with clear pathways to research translation and practical implementation to benefit communities with significant social disadvantages.

Our revised model for research co‐creation complements and builds on previous research related to co‐creation, cross‐sectoral collaboration and translational research. Components of our revised model are supported by both theoretical and empirical literature which highlights the importance of building and maintaining relationships of trust and ‘partnership synergy’ in collaborative research,^33^, ^41^, ^44^ the key role of local champions^47^, ^48^ and the need for resources to sustain such initiatives.^41^ Our model also advances from this foundation by focussing on practical stages, strategies and structures to build research partnerships and work towards co‐creation in real‐world health services and health systems. This manuscript is also one of few to report on collaborations specifically focused on health literacy. Another example is the Health Literacy Initiative involving Keele University and Stoke‐on‐Trent City Council Public Health.^44^ In describing their collaborative health literacy work, Estacio et al., for example, similarly noted the importance of trust to ensure that the partnership was sustainable and able to achieve systemic transformations. In their case, and our own experience, the growth and development of health literacy collaborations was based on mutual trust from individual members and the understanding that the partners were contributing to the achievement of a common goal.^44^

### 4.1 Strengths and limitations

We have developed a revised model of co‐creation based on our experience in establishing the Health Literacy Hub in western Sydney, Australia. Without testing in other settings, it is not yet clear whether this model is replicable or which components are entirely necessary for similar success. In addition, this model and manuscript may not capture the perceptions of all partners engaged with the Hub. Going forward, a more formalized evaluation including all partners will be valuable. This could, for example, replicate the evaluation of a UK public health collaborative, AVONet, which used a convergent parallel mixed‐methods design with quantitative surveys and qualitative semistructured interviews to capture the experiences of all partners involved in the collaboration in some way.^49^

## 5 CONCLUSIONS

A co‐creation approach—with its necessary time and resource commitments—has not always been rewarded in research. However, as researchers are pressed to highlight ‘impact’ by a growing number of funding bodies, co‐creation becomes more attractive. For the past 5 years, we have worked to build a practical and sustainable working relationship between an academic research lab and a local health system and its community focussed on improving health literacy. The goal was to improve both service delivery effectiveness and research output by leveraging combined resources, with involvement and input from all partners throughout the entire research journey. Our conceptual model reinforces core learnings from this process. We engaged broadly through the strategic development of a community of practice, with extensive commitments to build capability and relationships of trust and to progress research partnerships from relationship‐building activities to co‐creation. Partnership with local ‘champions’ and co‐generated resources helped to maintain momentum. Our co‐creation model can provide useful insight into mechanisms that have created an effective, collaborative and practical approach to researching and solving locally based problems working alongside healthcare staff and consumers.

## AUTHOR CONTRIBUTIONS

Danielle M. Muscat developed the revised conceptual model of co‐creation and drafted the manuscript. Don Nutbeam was a major contributor in writing the manuscript and revising the conceptual model. All authors played a key role in the establishment of the Health Literacy Hub and read and approved the final manuscript.

## CONFLICT OF INTEREST

D. M. M., K. M. and J. A. are joint Directors of Health Literacy Solutions Pty Ltd.; a health literacy consultancy company. The remaining authors declare no conflict of interest.

## Data Availability

Data sharing is not applicable to this article as no data sets were generated or analysed during the current study.
